# Deep-4mCGP: A Deep Learning Approach to Predict 4mC Sites in *Geobacter pickeringii* by Using Correlation-Based Feature Selection Technique

**DOI:** 10.3390/ijms23031251

**Published:** 2022-01-23

**Authors:** Hasan Zulfiqar, Qin-Lai Huang, Hao Lv, Zi-Jie Sun, Fu-Ying Dao, Hao Lin

**Affiliations:** School of Life Science and Technology, Center for Informational Biology, University of Electronic Science and Technology of China, Chengdu 610054, China; hasanzulfiqar66@gmail.com (H.Z.); huangqinlai2016@outlook.com (Q.-L.H.); hao.lyu@uzh.ch (H.L.); 15848174320@163.com (Z.-J.S.); N2107277C@e.ntu.edu.sg (F.-Y.D.)

**Keywords:** deep learning, alteration, features vector, genomics, algorithm

## Abstract

4mC is a type of DNA alteration that has the ability to synchronize multiple biological movements, for example, DNA replication, gene expressions, and transcriptional regulations. Accurate prediction of 4mC sites can provide exact information to their hereditary functions. The purpose of this study was to establish a robust deep learning model to recognize 4mC sites in Geobacter pickeringii. In the anticipated model, two kinds of feature descriptors, namely, binary and *k*-mer composition were used to encode the DNA sequences of *Geobacter pickeringii*. The obtained features from their fusion were optimized by using correlation and gradient-boosting decision tree (GBDT)-based algorithm with incremental feature selection (IFS) method. Then, these optimized features were inserted into 1D convolutional neural network (CNN) to classify 4mC sites from non-4mC sites in *Geobacter pickeringii*. The performance of the anticipated model on independent data exhibited an accuracy of 0.868, which was 4.2% higher than the existing model.

## 1. Introduction

Alterations in DNA play a significant role in gene expression and regulation, DNA replication, and transcriptional regulation. Methylcytosine is a key epigenetic trait at 5′-cytosine-phosphate-guanine-3′ site. Methylcytosine is precisely correlated with cell growth and chromosomal protection [1,2]. 5-Hydroxymethylcytosine (5hmC), 5-methylcytosine (5mC), and 4-methylcytosine (4mC) are the familiar cytosine methylations in multiple genomes of prokaryotes and eukaryotes [3,4]. 5mC is a frequent type of methylcytosine and responsible for many neurodegenerative and cancerous diseases [5]. 4mC is a significant alteration that protects genomic knowledge from weakening by restriction enzymes [6].

Precise identification of 4mC sites can give important signs to understand the method of gene regulation. At present, there are several techniques to recognize 4mC sites, for example, single-molecule real-time sequencing [7], mass spectrometry [8], and bisulfite sequencing [9], but these techniques are time-consuming and expensive when utilized on next-generation sequencing data. Hence, a computational model to identify 4mC sites is needed on an urgent basis. Currently, a few computational and mathematical methods have been introduced to predict 4mC sites in multiple species. In 2017, Chen at al. [10] introduced the first computational model to predict 4mC sites in multiple species on the basis of confirmed 4mC dataset. Subsequently, Wei at al. [11] designed the novel iterative feature illustrative algorithm for the prediction of 4mC sites. Tang et al. [12] introduced the new linear integration method by merging the existing models for the identification of 4mC sites. Afterwards, Manavalan et al. [13] established the new tool Meta-4mCpred to recognize 4mC sites in six different species. Khanal et al. [14] introduced the first deep learning model 4mCCNN by utilizing numerous feature combinations [15,16,17] for the prediction of 4mC sites in multiple genomes [18]. Although the prediction model 4mCCNN can yield good outcomes, there is still space for more improvement.

**Figure 1:**
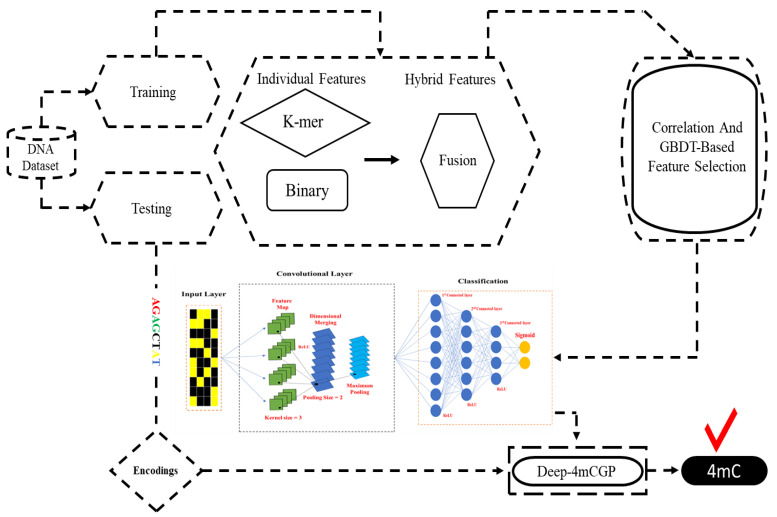
Flowchart of the whole study.

To tackle these hitches, we constructed a 1D CNN model to recognize 4mC sites in *Geobacter pickeringii*. Figure 1 illustrates the flowchart of the whole study. Binary and *k*-mer nucleotide composition descriptors were used to encode DNA sequences of *Geobacter pickeringii* into feature vectors and then these features were optimized by using a correlation and gradient-boosting decision tree (GBDT)-based algorithm with incremental feature selection (IFS) method. After this, these optimized features were inserted into 1D CNN-based classifier using 10-fold cross-validation and we attained the finest model to classify 4mC from non-4mC.

## 2. Results and Discussion

### 2.1. Performance Evaluation

**Figure 2:**
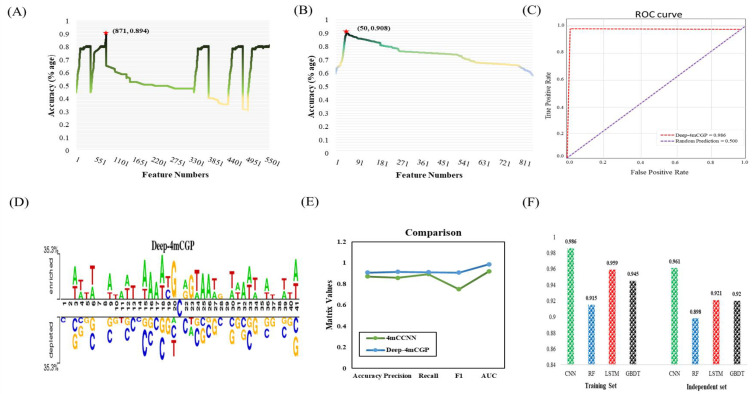
(**A**,**B**) The IFS technique for recognizing 4mC sites. Initially, 871 best features were picked from an overall 5624 by correlation measures (**A**). A total of 50 more optimized features were also attained from 871 best features by the using of GBDT on 10-fold CV. The Acc increases from 0.894 to 0.908 (**B**). Plot showing the *AUROC* curve of Deep-4mCGP on 10-fold CV (**C**). Nucleotides allocation along the alteration site (**D**). Performance comparison of Deep-4mCGP with 4mCCNN on 10-fold cross-validation (**E**). *AUROC* of predictors on training and independent data (**F**).

We constructed a 1D CNN-based model named Deep-4mCGP for the identification of 4mC sites in *Geobacter pickeringii.* In the first step, we converted the sequence data in to feature vectors by using *k*-mer nucleotide composition and binary encodings. Subsequently, these feature vectors were improved by means of correlation and GBDT-based algorithm with IFS method. Initially, correlation and then GBDT with IFS were utilized to pick the finest features. Figure 2A,B displays the IFS curve of top features. Afterward, these finest features were inserted into 1D CNN by using 10-fold cross-validation to classify 4mC sites from non-4mC sites in *Geobacter pickeringii.* In this work, 10-fold cross-validation was employed to examine the efficiency of the model. The data were arbitrarily divided into 10 segments of equal proportion. Each segment was independently tested by the model, which was trained on the outstanding nine segments. Thus, 10-fold cross-validation technique was executed 10 times, and the average of the outcomes was the ultimate result. *AUROC* of the anticipated model was 0.986, which was 6.5% higher than the existing model. The *accuracy, precision, recall,* and *F1* are shown in Table 1, and the *ROC* curve is shown in Figure 2C.

### 2.2. Sequence Composition Analysis

The pattern of sequence along the alteration site is a crucial phase to recognize and understand the definition of genomic disparities [19]. In this work, we utilized Two Sample Logo [20] to inspect the dispersal of nucleotides along the 4mC site. Figure 2D illustrates the dispersal of nucleotides. Nucleotides ‘A’ and ‘T’ were separately rich at the upstream and downstream of the positive sequences, e.g., five consecutive ‘A’ nucleotides (30–34) and four successive ‘A’ (15–18, 24–27) originated in positive sequences. Nucleotides ‘C’ and ‘G’ were abundant at the upstream and downstream of the negative sequences, e.g., five repeated ‘G’ nucleotides (30–34) and four repeated ‘G’ nucleotides (3–6, 24–27) and four consecutive ‘C’ nucleotides (15–18) were noticed in negative sequences. Figure 2D shows that there was a significant variance amongst 4mC sequences and non-4mC sequences. The consequences proposed that the dispersal of nucleotides in diverse places are supportive for the precise identification of 4mC.

### 2.3. Comparison on the Basis of Independent Data

Features fusion were inserted into LSTM [21], GBDT [22], and RF [23,24] to compare with the CNN-based model [25]. Ultimately, on the basis of *AUROC*, we achieved a perfect model for each predictor, which is shown in Table 1 and Figure 2F. Comparison of anticipated model with 4mCCNN by using 10-fold cross-validation is shown in Figure 2E. On the independent data (200 Pos. seq and 200 Neg. seq) the efficiency of Deep-4mCGP was checked and then compared with the existing 4mCCNN. The *accuracy, precision, recall, F1,* and *AUROC* of the 4mCCNN were 0.826, 0.818, 0.823, 0.825, and 0.920, respectively. The *accuracy, precision, recall, F1,* and *AUROC* of Deep-4mCGP were 0.868, 0.876, 0.773, 0.859, and 0.961, respectively. The performance of the anticipated Deep-4mCGP on independent data exhibited the accuracy of 0.868, which was 4.2% higher than the 4mCCNN. The performance comparison is shown in Table 2.

## 3. Materials and Methods

Authentic data are a significant requirement for the construction of a machine learning-based model [26,27]. Thus, we acquired the data of 1138 (569 Pos. seq and 569 Neg. seq) sequences of *Geobacter pickeringii* from the work of Chen et al. [10] for training and testing the model. Moreover, we attained the data of 400 sequences (200 Pos. seq and 200 Neg. seq) from the work of Manavalan et al. [13] for the sake of independent testing.

### 3.1. Feature Descriptors

Selecting useful and ideal features is an important step in developing machine learning models [4,28,29,30,31,32,33,34,35,36,37]. Converting the DNA sequences into numerical feature vectors is key in the recognition of functional elements, e.g., physiochemical properties, natural vectors, binary composition, and *k*-mer nucleotide compositions, which have been utilized in computational biology and bioinformatics [38,39]. In this study, binary and *k*-mer composition were used to encode DNA sequences of *Geobacter pickeringii.*

#### 3.1.1. *k-mer*

*k-mer* composition has the ability to show interactions between nucleotides of DNA sequences [40]. The residues of nucleotides can be attained by setting the size of window and steps. A random sample *F* with *n* sequence length can be designated as

(1) $$F=S_{1}\;S_{2}\;S_{3}\ldots ..S_{i}\ldots ..S_{\left(n-1\right)}\;S_{n}\;$$

where *S_i_* indicates the *i-*th nucleotide of the DNA sequences and can be converted in to 4*^k^* D features vector with the help of *k-mer.*

(2) $$F_{k}={\left[d_{1}^{k-tuple}d_{2}^{k-tuple}\ldots \ldots .d_{i}^{k-tuple}\ldots ..d_{4^{k}}^{k-tuple}\right]}^{T}$$

where *d_1_^k-tuple^* denotes the incidence of *i-*th *k-mer* and *T* represents the transposition. If the value of *k* is equal to 1, then DNA sequence will be decoded in to 4D features vector, and if the value of *k* is equal to 2, then DNA sequence will be 16D features vector. In this work, *k* was set as 1, 2, 3, 4, 5, 6. Consequently, DNA sequences were converted into (4^1^ + 4^2^ + 4^3^ + 4^4^ + 4^5^ + 4^6^ = 5460D) formulated as

(3) $$F=F_{1}\cup^{\;}\;F_{2}\cup^{\;}\;F_{3}\cup^{\;}\;F_{4}\;\cup^{\;}\;F_{5}\cup^{\;}\;F_{6}\;$$

#### 3.1.2. Binary

Binary encodings such as 0s and 1s have the ability to illustrate any information. Therefore, we can transform DNA sequence in the form of 0s and 1s. In this work, DNA sequences of *Geobacter pickeringii* with length of 41bp was encoded into the (4 × 41 = 164D) features vector.

### 3.2. Feature Selection

#### 3.2.1. Correlation

Correlation is a familiar comparison amongst two different features, e.g., if the features are un-correlated, then the correlation will be zero; otherwise, it will be *±*1. Two complete modules named classical linear correlation and correlation on the basis of information theory were implemented to compute the correlation amongst the two unique variables. Linear correlation coefficient is the most acquainted and utilizable. The linear correlation coefficient ‘*r*’ for a pair of (*p*, *q*) variables is specified as

(4) $$r=\frac{\sum^{\;}(p_{i}-{\bar{p}}_{i})(q_{i}-{\bar{q}}_{i})}{\sqrt{\sum^{\;}{\left(p_{i}-{\bar{p}}_{i}\right)}^{2}}\;\sqrt{\sum^{\;}{\left(q_{i}-{\bar{q}}_{i}\right)}^{2}}}$$

Correlation generates good results in smaller datasets, but the performance of correlation coefficient is not up to the mark on gigantic amounts of data. Therefore, it is necessary to determine the substantial relationship amongst the features. Thus, we utilized the *t*-test to investigate the statistical correlation between the features and picked the significant features. The value of ‘*t*’ can be computed as

(5) $$t=r\;\sqrt{\frac{n-2}{1-r^{2}}}$$

where ‘*r*’ signifies the coefficient of correlation and ‘*n*’ represents the occurrences. ‘*n*−2′ denotes the degree of freedom. Probability of the significance relation is 0.05. If ‘*t*’ is greater than the probability of the significance relation 0.05, then the feature will be selected.

#### 3.2.2. GBDT with IFS

GBDT is a popular machine learning-based classifier that has been utilized in various mathematical, cheminformatics, and bioinformatics tools [41,42]. It has the ability to establish a scalable and reliable prediction model by utilizing non-linear joints of weak learners [43].

(6) $$\{(x_{1},y_{1})\ldots (\;x_{n},y_{n})\}\;(∴\;x_{i}\epsilon \;x\;\subseteq \;S_{n},\;and\;y_{i}\epsilon \;y\;\subseteq \;S)\;\;q_{k\;}\left(x\right):=\sum_{k=1}^{k}D\;\left(x;\theta_{k}\right)$$

where $\theta_{k}$ is minimal risk of the decision tree and $D_{k}\left(x;\theta_{k}\right)$ is the decision tree.

(7) $$\hat{\theta_{k}}=argmin\;\sum_{i=1}^{n}P\;\left(y_{i},\;q_{k-1}\left(x\right)+D\;\left(x;\theta_{k}\right)\;\right)(∴P\;\mathrm{is}\;\mathrm{the}\;\mathrm{loss}\;\mathrm{function})$$

GBDT also computes the concluding evaluations in an advancing mode.

(8) $$q_{k}\left(x\right)=q_{k-1}\left(x\right)+D\;\left(x;\theta_{k}\right)$$

Negative gradient loss function $q_{k-1}\;$is applied for residual computation.

(9) $$S_{ki}\;=-{\left[\frac{\partial P\left(y_{i},q\left(x_{i}\right)\right)}{\partial q\left(y_{i}\right)}\right]}_{q\left(x\right)=q_{k-1}\left(x\right)}\;(∴\;i=1,2,3\ldots .n)$$

Hence, we trained the anticipated model through $S_{ki}\;$to compute the minimal risk $\theta_{k}$. This kind of trees rationally represents the relations between variables, e.g., plotting the input *X* into *J* fragments $S_{1}\;\ldots \;S_{J}\;$, and output is $Z_{J}$ for area $S_{J}$.

(10) $$D\left(x;\theta \right)=\overset{J}{\underset{j=1}{\sum }}z_{j}I\left(x_{j}\;\epsilon \;S_{j}\;\right)$$

The IFS [44,45] method was implemented in this work to pick the finest feature. IFS estimates the performance of the best *q*-ranked features repetitively for *q* ϵ (1, 2, 3, … *n*), where ‘*n*’ is the overall number of the features. IFS frequently stops at the first scrutiny of performance. In IFS, features were picked incrementally from a randomly taken initial feature and the finest result from several randomly re-instated IFS processes were outputted. A brief explanation of the IFS technique can be found in [46].
**Algorithms 1: Correlation and GBDT-based Feature Selection Algorithm****Input**: Training Data*: = Q (L_1_, L_2_, ……, L_k_, L_c_)* **Output***:* *Q_best_* **1st Round**   1   Begin   2   **for** *i = 1 to k*         **do**   3   *r* = calculate correlational coefficient (*L_i_*, *L_c_*)   4   **end**   5   *p = 0.05*   6   *ρ= 0*   (*⸫* if there is no correlation among the *F_i_* and *F_c_*)   7   **for** *i = 1 to k*        **do**   8   *t =* to calculate the significance (*r,*
*ρ*) for *L_i_* (*⸫* by utilizing the *t-*test value from Equation (5))   9   **if**   *t >* critical value   10  *Q_best_ = Q list*   11  **end**   12  return *Q_best_* **2nd Round** **Input**:    *Q_best_: =*
${\left(x_{i},y_{i}\right)}_{i=1}^{n}\;$       Where, ($x_{i}$ = *data and*
$y_{i}$
*= label*)       *LF: = P (*$y_{i}$*, q (*x*))*  13   By initializing the model  14 $\;q_{{}^{\circ}}\left(x\right)$: = argument minimum $\sum_{i=1}^{n}P\left(y_{i},z\right)$  15   **for**
*I =* {*1, 2, 3, 4, 5…, n*}          **do**  16   **for**
*k =* {*1, 2, 3, 4, 5…, K*}           **do**  17   Pseudo residual error calculations:  $S_{ki}\;$*=*$\;-{\left[\frac{\partial P\left(y_{i},q\left(x_{i}\right)\right)}{\partial q\left(y_{i}\right)}\right]}_{q\left(x\right)=q_{k-1}\left(x\right)}$  18   **end**  19   **end**  20   On the basis of $S_{ki}\;$, $\theta_{k}$*=* {$S_{kj}j=\left[1,2,3\ldots J\right]$}, we built a decision tree $D_{k}\left(x;\theta_{k}\right)$  21   **for** *j =* {*1, 2, 3, 4, 5…., J*}            **do**  22   $z_{kj}$
*=* argument minimum $\sum_{x_{i}\in \;S_{kj}}^{n}P\left(y_{i},q_{k-1}\left(x\right)+z\right)$  23   **end**  24   Updating the model $q_{k}\left(x\right)$
*=*
$q_{k-1}\left(x\right)$
*+*
$\sum_{j=1}^{j}z_{kj}I\left(x\hat{I}S_{kj}\right)$  25   *q (x) =*
$\sum_{k=1}^{K}\sum_{j=1}^{J}z_{kj}I\left(x\hat{I}S_{kj}\right)$ **Output**: The decision tree function  *q (x)*

### 3.3. Convolutional Neural Network

LeCun at al. [47] introduced convolutional neural network, and now it has been roughly utilized in many biological and bioinformatics advances [48,49,50]. The fundamental principle of CNN is to create abundant filters that have the ability to produce hidden topological features from data by executing pooling procedures and layer-wise convolutions. The performance of CNN on 2D data of images and matrices is exceptional [51]. Subsequently, 1D CNN has been used to tackle the difficulties of biomedical sequence data identification and the research associated with natural language processing [41,52]. In this work, we implemented 1D CNN to identify 4mC sites in *Geobacter pickeringii.* We employed Keras 2.3.1 [53], TensorFlow 2.1.0, and Python 3.5.4 to perform this experiment. The best tuning parameters are recorded in Table 3.

### 3.4. Metrics Evaluation

Precision, accuracy, recall, and F1 [54,55,56] were employed to examine the effectiveness of the anticipated prediction model and formulated as

(11) $$\left\{ \begin{array}{l} Precision=\frac{TP}{TP+FP}\\ Recall=\frac{TP}{TP+FN}\\ Accuracy=\frac{TP+TN}{TP+FP+TN+FN}\\ F1=2\times \frac{Precision\times Recall}{Precision+Recall}\; \end{array}\right.$$

where ‘*TP*’ symbolizes the accurately predicted 4mC sequences, ‘*TN*’ represents the perfectly predicted non-4mC sequences, ‘*FP*’ indicates the non-4mC sequences predicted as 4mC sequences, and ‘*FN*’ indicates the 4mC sequences predicted as non-4mC sequences.

## 4. Conclusions

4mC is a type of DNA alteration that has the ability to synchronize multiple biological movements for example DNA replication, gene expressions, and transcriptional regulations. Accurate prediction of 4mC sites can provide exact information to their hereditary functions. Currently, several machine learning models have been used to predict 4mC sites in multiple genomes [10,12,13,57,58,59,60]. However, there is only one deep learning-based model, 4mCCNN [14], that exists for *Geobacter pickeringii*. In this work, a deep learning model was constructed to recognize 4mC sites in *Geobacter pickeringii*. In the anticipated model, two kinds of feature descriptors, namely, binary and *k*-mer composition were used to encode the DNA sequences of *Geobacter pickeringii*. The obtained features from their fusion were optimized by using correlation and GBDT-based algorithm with IFS method. Then, these optimized features were inserted into a 1D CNN-based classifier using 10-fold cross-validation, and we attained the finest model to classify 4mC from non-4mC. The performance of the anticipated Deep-4mCGP on independent data exhibited an accuracy of 0.868, which was 4.2% higher than the 4mCCNN. The source code and data are available at GitHub: https://github.com/linDing-groups/Deep-4mCGP (accessed on 19 January 2022). In future work, we have a plan to release a web-based application to make our anticipated model more convenient for the users without programming and statistical knowledge.

## Figures and Tables

**Table 1:** Outcomes of single encodings and their fusion based-models on training and independent data by using different classification algorithms. Bold is used to highlight the best results.

			Training Data					Independent Data				
Algorithm	FS	Method	*Accuracy*	*Precision*	*Recall*	*F1*	*AUROC*	*Accuracy*	*Precision*	*Recall*	*F1*	*AUROC*
LSTM	5460	*k*-mer	0.861	0.872	0.861	0.811	0.943	0.825	0.820	0.812	0.819	0.882
	164	Binary	0.834	0.828	0.837	0.838	0.875	0.801	0.804	0.798	0.801	0.872
	5624	Fusion	0.868	0.865	0.859	0.862	0.937	0.810	0.814	0.808	0.813	0.902
	871	Fusion	0.859	0.857	0.847	0.857	0.925	0.808	0.801	0.807	0.800	0.876
	50	Fusion	0.884	0.878	0.881	0.879	0.959	0.841	0.842	0.839	0.842	0.921
RF	5460	*k*-mer	0.831	0.862	0.758	0.664	0.936	0.809	0.838	0.761	0.648	0.909
	164	Binary	0.772	0.763	0.755	0.770	0.863	0.753	0.748	0.753	0.756	0.832
	5624	Fusion	0.844	0.847	0.839	0.845	0.891	0.795	0.788	0.783	0.794	0.887
	871	Fusion	0.847	0.849	0.851	0.846	0.897	0.801	0.800	0.800	0.798	0.878
	50	Fusion	0.866	0.858	0.861	0.854	0.915	0.812	0.808	0.814	0.812	0.898
GBDT	5460	*k*-mer	0.848	0.881	0.776	0.676	0.962	0.828	0.861	0.770	0.669	0.931
	164	Binary	0.827	0.821	0.823	0.827	0.895	0.782	0.778	0.779	0.781	0.862
	5624	Fusion	0.835	0.832	0.830	0.832	0.893	0.786	0.780	0.786	0.786	0.882
	871	Fusion	0.851	0.853	0.848	0.854	0.901	0.814	0.810	0.815	0.810	0.893
	50	Fusion	0.875	0.874	0.868	0.860	0.945	0.836	0.835	0.830	0.841	0.920
CNN	5460	*k-*mer	0.880	0.879	0.887	0.880	0.949	0.848	0.844	0.841	0.845	0.927
	164	Binary	0.868	0.836	0.834	0.832	0.928	0.798	0.802	0.807	0.790	0.881
	5624	Fusion	0.868	0.865	0.859	0.862	0.937	0.810	0.814	0.808	0.813	0.903
	871	Fusion	0.894	0.877	0.897	0.889	0.955	0.846	0.845	0.841	0.838	0.920
	50	Fusion	**0.908**	**0.914**	**0.910**	**0.908**	**0.986**	**0.868**	**0.876**	**0.773**	**0.859**	**0.961**

**Table 2:** Performance comparison of Deep-4mCGP with 4mCCNN.

Predictor	*CV*	*Accuracy*	*Precision*	*Recall*	*F1*	*AUROC*	Reference
4mcCNN	10 (folds)	0.871	0.857	0.893	0.750	0.921	[14]
Deep-4mCGP	10 (folds)	0.908	0.914	0.910	0.908	0.986	Deep-4mCGP
4mcCNN	Test (Ind)	0.826	0.818	0.823	0.825	0.920	[14]
Deep-4mCGP	Test (Ind)	0.868	0.876	0.773	0.859	0.961	Deep-4mCGP

**Table 3:** Program in TensorFlow 2.1.0 with employed parameters.

Classifier	Parameters
RF	N-estimators = 100, Learning-rate = 0.001, Mean absolute error = 0.143, Mean square error = 0.220
GBDT	N-estimators = 120, Learning-rate = 0.01, Mean absolute error = 0.117, Mean square error = 0.212
LSTM	nn.LSTM(input_size = feature_size, hidden_size = 128) nn.Linear(int_features = 128, out_features = 1) nn.Sigmoid() learning-rate = 0.001, Epoch = 100, Batch-size = 32
CNN	nn. Conv1d (in_channels = feature size, out_channels = 32, padding = valid, strides = 1, kernel_size = 2) nn.ReLU() nn.MaxPool 1d (padding = valid, strides = 2, pool_size = 2) nn. Dropout (*p* = 0.5) nn.Sigmoid() Learning-rate = 0.01, epoch = 80, batch-size = 32

## Data Availability

All the data are available at https://github.com/linDing-groups/Deep-4mCGP (accessed on 19 January 2022).
